# Distinctive Alterations of Functional Connectivity Strength between Vascular and Amnestic Mild Cognitive Impairment

**DOI:** 10.1155/2021/8812490

**Published:** 2021-05-19

**Authors:** Hui Li, Shuai Gao, Xiuqin Jia, Tao Jiang, Kuncheng Li

**Affiliations:** ^1^Department of Radiology, Beijing Chaoyang Hospital, Capital Medical University, Beijing 100020, China; ^2^Department of Radiology, Xuanwu Hospital, Capital Medical University, Beijing 10053, China; ^3^Beijing Key Lab of MRI and Brain Informatics, Beijing 10053, China

## Abstract

Widespread structural and functional alterations have been reported in the two highly prevalent mild cognitive impairment (MCI) subtypes, amnestic MCI (aMCI) and vascular MCI (VaMCI). However, the changing pattern in functional connectivity strength (FCS) remains largely unclear. The aim of the present study is to detect the differences of FCS and to further explore the detailed resting-state functional connectivity (FC) alterations among VaMCI subjects, aMCI subjects, and healthy controls (HC). Twenty-six aMCI subjects, 31 VaMCI participants, and 36 HC participants underwent cognitive assessments and resting-state functional MRI scans. At first, one-way ANCOVA and *post hoc* analysis indicated significant decreased FCS in the left middle temporal gyrus (MTG) in aMCI and VaMCI groups compared to HC, especially in the VaMCI group. Then, we selected the left MTG as a seed to further explore the detailed resting-state FC alterations among the three groups, and the results indicated that FC between the left MTG and some frontal brain regions were significantly decreased mainly in VaMCI. Finally, partial correlation analysis revealed that the FC values between the left MTG and left inferior frontal gyrus were positively correlated with the cognitive performance episodic memory and negatively related to the living status. The present study demonstrated that different FCS alterations existed in aMCI and VaMCI. These findings may provide a novel insight into the understanding of pathophysiological mechanisms underlying different MCI subtypes.

## 1. Introduction

Mild cognitive impairment (MCI), associated with deficits in multiple cognitive domains without notable affection on daily activities, is regarded as a risk state for dementia [1]. The two highly prevalent subtypes, amnestic MCI (aMCI) and vascular MCI (VaMCI), are considered the prodromal stage of Alzheimer's disease (AD) and vascular dementia (VD), respectively [2, 3]. It has been a notion that VaMCI subjects exhibit significant impairment in executive function and semantic memory while aMCI subjects show predominant deficits in episodic memory [4].

Magnetic resonance imaging (MRI) is an important imaging method for pathophysiological mechanism investigation and differential diagnosis of MCI subtypes. Widespread structural abnormalities and functional alterations have been reported in both VaMCI and aMCI groups [5–8]. Gray matter atrophies are mainly distributed in frontal and temporal brain regions, posteromedial cortices, and several subcortical brain sites, especially the medial temporal lobe, including the hippocampus and entorhinal cortex [9]. Using diffusion MRI scanning, microstructural deteriorations within the corpus callosum, capsule, periventricular white matter, cingulum, and occipitofrontal fasciculi have been reported [10–12]. Yu et al. reported significant differences in the relationship between fractional anisotropy (FA) and the Auditory Verbal Learning Test (AVLT) between the VaMCI and aMCI groups [13]. Functional connectivity (FC) changes are predominantly found in the default mode network and the medial temporal lobe [5, 6, 14]. Both decreased and increased brain activities related to the severity of cognitive decline have been demonstrated. Graph theory-based network analyses also reveal significant dysregulation of the topological organization of functional brain networks [15].

Recently, based on the graph theory, the human brain is considered a complex, interconnected network with a set of nodes linked by connections to support efficient information processing and integration [16]. Converging evidences in both structural [17] and functional network analyses [18] have demonstrated that some specific nodes with a large number of connections (or large degree) [19] play critical roles in fast information integration and communication with minimal energy cost [20]. Previous studies have investigated the changing pattern of functional network centrality by calculating functional connectivity strength (FCS) in some diseases such as social anxiety disorder and provide a novel insight into the understanding of underlying pathophysiological mechanisms [21]. Although previous studies have demonstrated altered FC in different MCI subtypes, the changing pattern in FCS remains unclear.

In the present study, we detected the differences in FCS among VaMCI subjects, aMCI subjects, and healthy control (HC) participants. Then, seed-based connectivity analysis were performed to further explore the detailed resting-state FC alterations, using the clusters showing significant differences in FCS as the seeds. We assumed that these MCI subtypes would show distinct FCS and FC changes within their signature large-scale networks as compared to HC. Further, we would explore how these changes relate to neuropsychological deficits in VaMCI and aMCI.

## 2. Materials and Methods

### 2.1. Participants

The study was conducted under a research protocol approved by the Ethics Committee of Beijing Xuanwu Hospital. Written informed consent was obtained from all participants prior to the study.

Twenty-six aMCI subjects, 31 VaMCI subjects, and 36 HC participants were recruited from Xuanwu Hospital in Beijing between 2017 and 2019. We followed the methods of Li et al. [22]. All participants underwent detailed medical history collection, physical examination, and neuropsychological evaluation by experienced neurologists that have been trained to unify the evaluation criteria. Neuropsychological assessments included Clinical Dementia Rating (CDR), Mini Mental State Examination (MMSE), Montreal Cognitive Assessment (MoCA), and activities of daily living (ADL).

The diagnosis of aMCI followed the criteria stipulated in 2011 by the National Institute on Aging and the Alzheimer's Association [3]. Other two conditions were satisfied as follows: (a) CDR score of 0.5 and a score of at least 0.5 on the memory domain [23] and (b) medial temporal lobe or hippocampal atrophy on MRI [24, 25].

The criteria used for the selection for the HC were as follows: (a) no complaints of cognitive changes, (b) no current or previous diagnosis of any neurological or psychiatric disorders, (c) no neurological deficiencies in physical examinations, (d) no abnormal neurological findings on brain MRI, and (e) CDR score of 0.

The inclusion criteria for the VaMCI were based on the *Diagnostic and Statistical Manual of Mental Disorders* (DSM), fourth edition, for VaMCI [26] including the following: (a) complaint of cognitive impairment, at least at one cognitive domain; (b) objective evidence for cognitive decline; (c) the clinical characteristics being consistent with the vascular etiology; (d) history of cerebrovascular disease, physical examination, and/or neuroimaging evidence for cerebrovascular disease; (c) exclusion of other possible diseases; and (d) ability to maintain independence in daily activities.

Participants were excluded if they had a history of psychiatric disorder and neurological conditions affecting cognition, such as head injury, depression, alcohol use disorder, epilepsy, and Parkinson's disease. Additional exclusion criteria included major medical illness, severe visual or hearing loss, and contraindications for MRI.

### 2.2. Magnetic Resonance Imaging Procedures

The subjects were scanned using a specific 3-Tesla GE scanner (General Electric, MRI750W, America). All participants were asked to remain still, stay awake, and keep their eyes closed. Head motion and scanner noise were reduced using foam padding and earplugs. All subjects underwent clinical standardized axial T2, axial fluid-attenuated inversion recovery (FLAIR), sagittal T1, and resting-state funcional MRI(fMRI)scans using an echo-planar imaging (EPI) sequence. The following parameters were used for fMRI images for aMCI and HC participants: repetition time (TR) = 2000 ms, echo time (TE) = 30 ms, flip angle (FA) = 90°, field of view (FOV) = 256 × 256 mm^2^, data matrix = 64 × 64, 36 axial slices, slice thickness/gap = 3/1 mm, and number of repetitions = 180. Meanwhile, the following parameters were used for fMRI images for VaMCI subjects: TR = 2000 ms, TE = 30 ms, FA = 90°, FOV = 220 × 220 mm^2^, slice thickness/gap = 3.6/0.4 mm, 36 axial slices, data matrix = 64 × 64, and number of repetitions = 185. Parameters for axial T2 were as follows: TR = 4581 ms, TE = 82 ms, FOV = 220 × 220 mm^2^, slice thickness/gap = 5.5/1.0 mm, data matrix = 416 × 416, and 20 axial slices. Parameters for sagittal T1 were as follows: TR = 1750 ms, TE = 24 ms, FOV = 240 × 216 mm^2^, slice thickness/gap = 5.5/1.0 mm, data matrix = 288 × 224, and 20 axial slices. Parameters for axial FLAIR were as follows: TR = 7000 ms, TE = 120 ms, FOV = 220 × 220 mm^2^, slice thickness/gap = 5.5/1.0 mm, data matrix = 416 × 416, and 20 axial slices.

### 2.3. MRI Data Preprocessing

Functional image data were preprocessed using the Data Processing Assistant for Resting-State fMRI (DPARSF) software package v4.5 [27] and Statistical Parametric Mapping 12 based on MATLAB 2013a. The first 5 images of each fMRI dataset were discarded to reduce the initial fluctuation of MRI signals in aMCI and HC, while the first 10 images of each fMRI dataset were discarded in VaMCI. The numbers of remaining images were all the same (175 images) in the three groups. Briefly, the fMRI time series were first corrected for within-scan acquisition time differences between slices and realigned to correct for head motion. The participants with head movement exceeding 2.0 mm of translation or 2.0° of rotation in any direction were excluded. In addition, one-way analysis of variance showed that there was no significant difference in the values of the mean framewise displacement (FD) among the three groups. All the realigned images were spatially normalized to the Montreal Neurological Institute EPI template, and each voxel was resampled to 3 × 3 × 3 mm^3^. To avoid introducing artificial local spatial correlations, the images were not smoothed [28]. Denoising steps included linear detrending and regression of the six motion parameters and their first-order derivatives and regression of white matter and cerebrospinal fluid (CSF) [29]. Then, the time series were temporally band-pass filtered (0.01–0.08 Hz) to reduce the effects of high-frequency physiological noises.

### 2.4. Network Analysis

For each voxel, the time series was extracted and Pearson's correlation coefficients between the time series of the voxel and all other voxels' time series were calculated within a gray matter mask (*N* voxels = 58,108). Then, the correlation coefficients greater than 0.2 were averaged over the gray matter mask, and a 3D FCS map for each subject was obtained [30]. Finally, the FCS map was converted to *z* scores and smoothed with a 6 mm full width at half-maximum Gaussian kernel.

## 3. Statistical Analyses

### 3.1. Demographic and Neuropsychological Variables

For gender assessment, *x*^2^ tests were used. The age, education level, and cognitive performance differences among the three groups were estimated by one-way analysis of variance. These analyses were implemented in SPSS 21.

### 3.2. Group Differences in Functional Connectivity Strength

One-sample *t*-tests were performed to identify the patterns of FCS within each group [21], and the significant threshold was set at voxel-level *p* < 0.05 corrected for multiple comparisons using the family-wise error rate (FWE). To find the altered FCS regions, one-way analysis of covariance (ANCOVA) was then performed to compare the FCS maps among the three groups, with age, gender, and education as covariates. The significant threshold was set at cluster-level *p* < 0.05, FWE corrected. The FCS values of the regions showing significant group difference were extracted for *post hoc* pairwise comparisons and partial correlations. *Post hoc* pairwise comparisons were conducted by independent-sample *t*-tests to compute the differences between any two groups using SPSS 21.

### 3.3. Seed-Based Resting-State Functional Connectivity Analysis

Seed-based connectivity analysis were performed to further explore the detailed resting-state FC alterations, using the clusters showing significant differences in FCS as the seeds. One-way ANCOVA was performed on the FC maps for each identified seed. The significant threshold was set at voxel-level *p* < 0.05, FWE corrected, with age, gender, and years of education as covariates. The FC values of the regions showing significant group differences were extracted for *post hoc* pairwise comparisons and partial correlations.

### 3.4. Relationships between Regional Connectivity Measures and Cognitive Performance

Partial correlation analysis was performed to explore the relationship between the connectivity measurements (i.e., FCS and seed-based FC) and clinical variables in VaMCI and aMCI separately, with age, gender, and years of education as covariates. Statistical significance was set at *p* < 0.05.

## 4. Results

### 4.1. Demographics and Clinical Characteristics of the Participants

The demographic and clinical data are shown in Table 1. Significant differences were found in cognitive assessments among the three groups while age, gender, and education years were matched well. Both MCI subtype groups showed lower scores in MMSE (*p* < 0.001) and MoCA (*p* < 0.001) and higher CDR scores (*p* < 0.001), indicating significant general cognitive decline as compared to HC. Meanwhile, both MCI subtype groups showed higher ADL scores (*p* < 0.001). In addition, the aMCI group showed lower cognitive assessment scores and higher ADL scores than VaMCI.

### 4.2. Within-Group FCS Analyses

The results derived from one-sample *t*-tests for the three groups are separately shown in Figures S1–S3. Functional nodes with a large number of connections were found mainly in the temporal and parietal brain regions, as well as several occipital and frontal cortices.

### 4.3. Between-Group FCS Analyses

**Figure 1:**
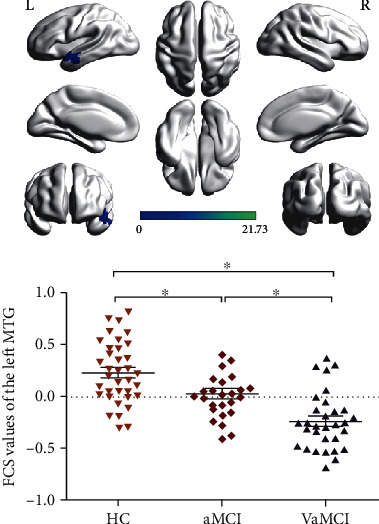
The result of one-way ANCOVA and *post hoc* analysis indicated significant decreased FCS in the left MTG among the VaMCI, aMCI, and HC. ∗ means *p* < 0.05 in the two-sample *t*-test using SPSS21. L: left; R: right; MTG: middle temporal gyrus; FCS: functional connectivity strength; VaMCI: vascular mild cognitive impairment; aMCI: amnestic mild cognitive impairment; HC: healthy control.

One-way ANCOVA indicated significant FCS difference in the left middle temporal gyrus (MTG) among the three groups (Table 2, Figure 1).

*Post hoc* analysis revealed that (1) as compared to HC, subjects with aMCI showed significant decreased FCS in the left MTG; (2) as compared to HC, subjects with VaMCI showed significant decreased FCS in the left MTG; and (3) as compared to aMCI, subjects with VaMCI showed significant decreased FCS in the left MTG (Figure 1).

### 4.4. Alterations in Seed-Based Resting-State Functional Connectivity

**Figure 2:**
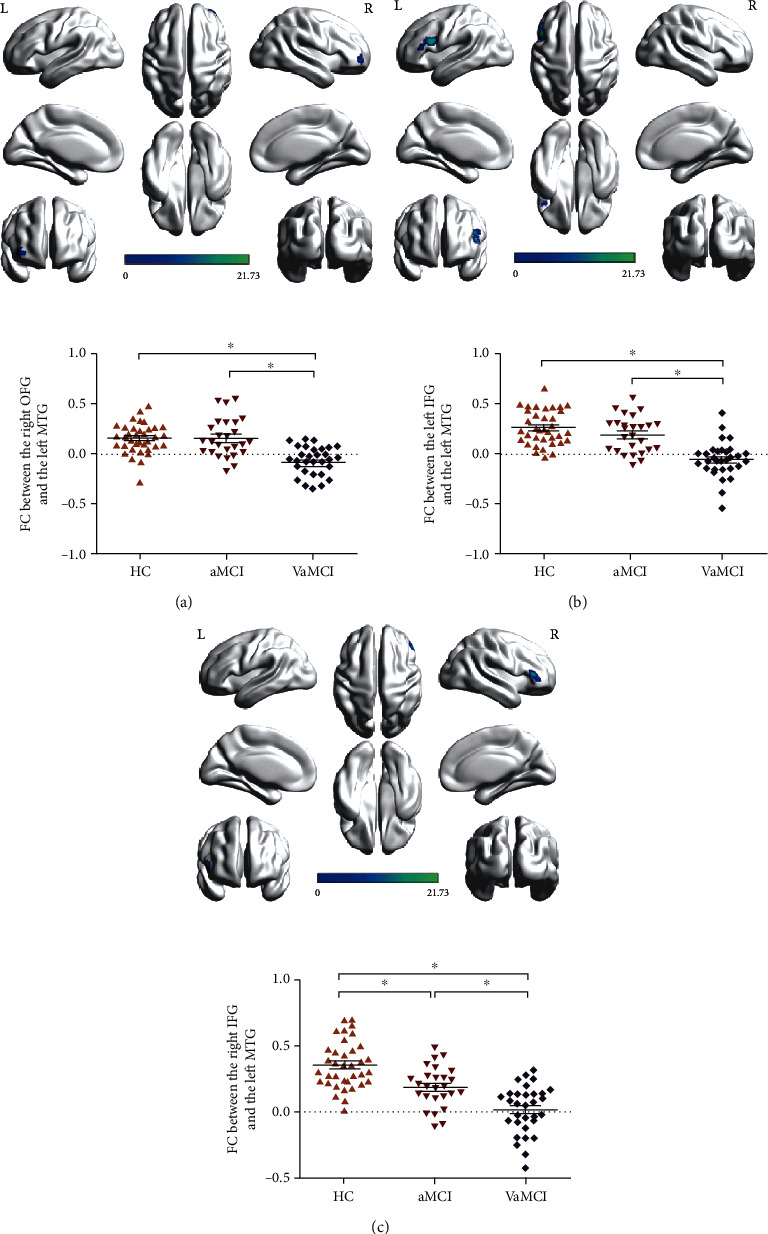
The results of one-way ANCOVA and *post hoc* analysis indicated significant decreased FC between the left MTG and the right OFG (a), the left IFG (b), and the right IFG (c) among the three groups. ∗ means *p* < 0.05 in the two-sample *t*-test using SPSS21. L: left; R: right; FC: functional connectivity; MTG: middle temporal gyrus; OFG: orbital frontal gyrus; IFG: inferior frontal gyrus; VaMCI: vascular mild cognitive impairment; aMCI: amnestic mild cognitive impairment; HC: healthy control.

For further detailed analysis regarding the left MTG FC pattern, the subsequent seed-based FC analysis revealed that the left MTG network in HC was composed of the medial frontal, temporal cortical, and parietal sites. However, the frontal sites were excluded in both aMCI and VaMCI (Figures S4–S6). One-way ANCOVA indicated significant FC differences between the left MTG and the right orbital frontal gyrus (OFG), the left inferior frontal gyrus (IFG), and the right IFG among the three groups. *Post hoc* analysis revealed that significant FC disruptions in the right OFG and the left IFG were found only in VaMCI, while significant reduced FC in the right IFG were found in both aMCI and VaMCI with more serious disruption in the VaMCI group (Table 2, Figure 2).

### 4.5. Correlations between Cognitive Performances and Connectivity Measures

**Figure 3:**
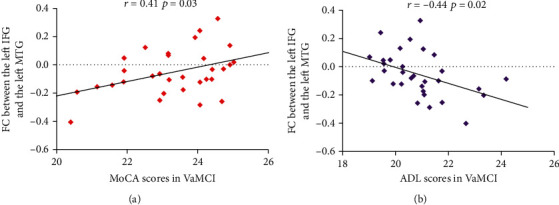
(a) The FC values between the left MTG and left IFG were positively correlated with the cognitive performance episodic memory measured by MoCA scores. (b) The FC values between the left MTG and left IFG were negatively correlated with ADL scores. FC: functional connectivity; MTG: middle temporal gyrus; IFG: inferior frontal gyrus; MoCA: Montreal Cognitive Assessment; ADL: activities of daily living; VaMCI: vascular mild cognitive impairment.

Partial correlation analysis revealed that the FC values between the left MTG and left IFG were positively correlated with the cognitive performance episodic memory measured by MoCA scores (*r* = 0.41, *p* = 0.03, Figure 3(a)) and negatively correlated with ADL scores (*r* = −0.44, *p* = 0.02, Figure 3(b)) in the VaMCI group.

## 5. Discussion

Based on resting-state fMRI and graph theory approaches, the present study reported decreased FCS in the left MTG in both VaMCI subjects and aMCI subjects, especially in the VaMCI group. Furthermore, more frontal regions showed disrupted resting-state FC to left MTG in the VaMCI group than in the aMCI group. These aberrant brain connectivities may be the neurobiological mechanism underlying the cognitive deficits.

Based on the computational analysis of anatomic connectivity, Sporns et al. formally defined the cortex nodes that have disproportionately numerous connections as hubs [31]. Network analysis studies using postmortem tracing techniques in nonhuman primates [32], vivo tract-tracing [33], and fMRI in humans [34] further confirmed the existence of hubs. An accurate reference map of prominent cortical hubs consisting of posterior cingulate, lateral temporal, lateral parietal, and medial/lateral prefrontal cortices had been obtained, and the left MTG is one of the peak locations of the largest 10 hubs [35].

MTG plays an important role in verbal short-term memory [36] and global cognitive function [37]. Previous studies have shown that MTG is one of the most vulnerable brain regions affected by cognition impairment-related pathological changes. In voxel-based morphometric study, significant gray matter volume reductions in MTG have been reported in both patients with aMCI and VaMCI [38–40]. As for the whole-brain function network, an altered FC pattern and FC density in MTG have been reported by fMRI [5, 41, 42]. In addition, significantly decreased amplitude of low-frequency fluctuation (ALFF) in the left MTG had been reported in VaMCI [43]. In the present study, the left MTG was found to exhibit decreased FCS in the aMCI and VaMCI groups. Furthermore, compared with aMCI, the VaMCI group showed more serious FCS reduction in MTG. These results suggested that, as a critical network node with rich connections, the MTG was preferentially targeted in cognition impairment-related diseases, especially in the VaMCI group.

Subsequently, we made the left MTG-based whole-brain FC maps for further analysis. Notably, reduced FC between the left MTG and some frontal brain regions, including the right OFG and bilateral IFG, were mainly reported in the VaMCI group. Previous studies indicated that the small vessel disease-related cognitive impairments, including the deterioration of psychomotor speed, executive control, and global cognitive function, may be related to disruption and disconnection of the frontal-subcortical pathways [44]. Both IFG and OFG were associated with the executive function [45, 46]. This is consistent with the notion that the dorsolateral prefrontal circuit is involved mainly in executive function [47, 48]. Using fMRI, decreased ALFF and FC in the frontal lobe have been repeatedly reported in VaMCI [49–52]. In addition, orbitofrontal syndromes, such as disinhibition and decreased social behavior, are more frequent in VaMCI than in aMCI. In the present study, disrupted FC between the frontal lobe and MTG was found in the MCI group, especially the VaMCI group. Furthermore, FC values between the left MTG and left IFG were associated with cognitive and living status, each reflected by the MoCA and ADL scores in VaMCI patients, which may be related to the executive dysfunction.

Several limitations need to be considered for this study. First, the sample size of the study was relatively small, and studies with a larger sample will be needed to replicate the current findings. Second, there were some subtle differences between the two MRI sequences. Unified MRI sequence parameters should be used in the future study.

In conclusion, the present study demonstrated for the first time that the MCI was associated with disrupted functional brain networks and significantly decreased FCS in the MTG. Furthermore, disrupted FC between the frontal lobe and MTG was found in the VaMCI group, which may be related to the executive dysfunction. These findings may open a novel way to better understand the pathophysiological mechanisms of MCI.

## Figures and Tables

**Table 1:** Clinical characteristics of subjects with aMCI and VaMCI and HC.

Characteristics	HC (*n* = 36)	VaMCI (*n* = 31)	aMCI (*n* = 26)	Group *p*
Age (years)	64.22 ± 6.97	64.93 ± 10.11	66.04 ± 7.92	0.62
Gender, M/F	17/19	18/13	8/18	0.11
Education (years)	10.72 ± 5.41	9.00 ± 2.00	10.77 ± 4.65	0.08
CDR	0	0.5	0.5	<0.001
MMSR	28.13 ± 2.77	26.32 ± 2.05	23.73 ± 3.85	<0.001
MoCA	25.22 ± 2.89	23.32 ± 1.32	19.81 ± 4.71	<0.001
ADL	20.00 ± 0.00	20.87 ± 1.43	22.84 ± 2.78	<0.001

Values represent means ± SD; *p* values were derived from the one-way NOVA test comparing the three groups, except for “gender” where the *p* value was obtained using the *x*^2^ test. MMSE: Mini Mental State Examination; MoCA: Montreal Cognitive Assessment; ADL: activities of daily living; VaMCI: vascular mild cognitive impairment; aMCI: amnestic mild cognitive impairment; HC: healthy control.

**Table 2:** FCS differences and left MTG FC differences among subjects with aMCI and VaMCI and HC.

Brain regions	Cluster size	MNI			*F*-score
		*x*	*y*	*z*	
FCS					
Lt. MTG	189	-60	-3	-21	9.823
FC					
Rt. OFG	26	33	48	-3	20.8862
Rt. IFG	50	57	33	6	27.3006
Lt. IFG	98	-45	18	18	27.7231

Lt.: left; Rt.: right; FC: functional connectivity; MTG: middle temporal gyrus; OFG: orbital frontal gyrus; IFG: inferior frontal gyrus; VaMCI: vascular mild cognitive impairment; aMCI: amnestic mild cognitive impairment; HC: healthy control; MNI: Montreal Neurological Institute.

## Data Availability

The datasets analyzed during the current study are not publicly available due to the unfinished study of the whole project but are available from the corresponding author on reasonable request.

## Supplementary Materials

Supplementary Materials The attached file of supplementary materials presents the results derived from one-sample *t*-tests of within-group analysis for the three groups. Figures S1–S3 show the functional nodes with a large number of connections in HC, aMCI, and VaMCI, separately. Figures S4–S6 show the whole-brain FC of the left MTG in HC, aMCI, and VaMCI, separately.
