# Supplementary material for: Distinctive Alterations of Functional Connectivity Strength between Vascular and Amnestic Mild Cognitive Impairment
Source: Neural Plast. 2021 May 19;2021:8812490. doi: 10.1155/2021/8812490 (PMC8159649; doi:10.1155/2021/8812490)

**Supplementary materials**

**Figure S1**. Functional nodes with large number of connections in health control group. The threshold was set at voxel level *p* < 0.05 corrected for multiple comparisons using the family-wise error rate. The color bar represents the t value of the within-group analysis. L, left; R, right.


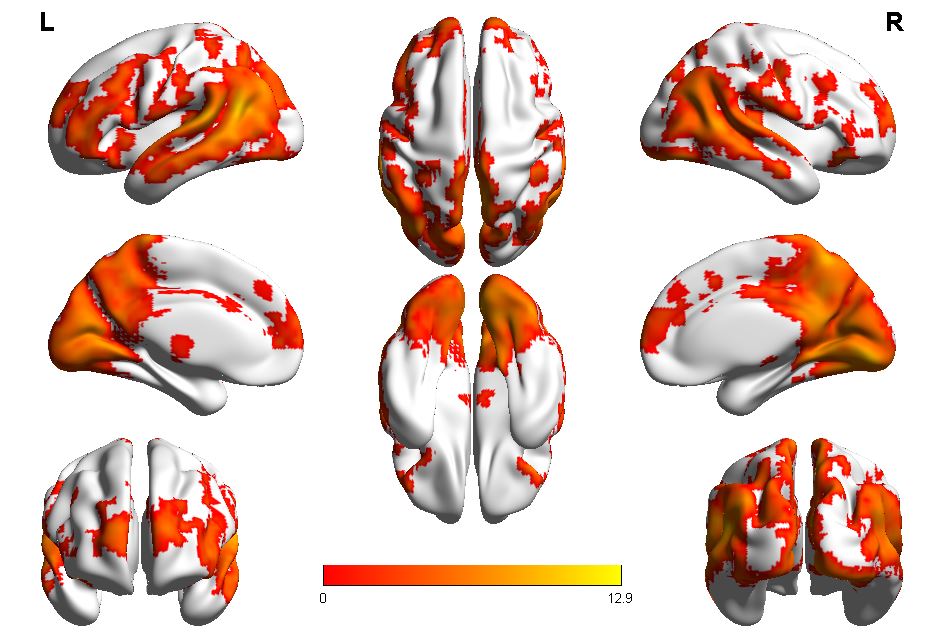


**Figure S2**. Functional nodes with large number of connections in amnestic mild cognitive impairment group. The threshold was set at voxel level *p* < 0.05 corrected for multiple comparisons using the family-wise error rate. The color bar represents the t value of the within-group analysis. L, left; R, right.


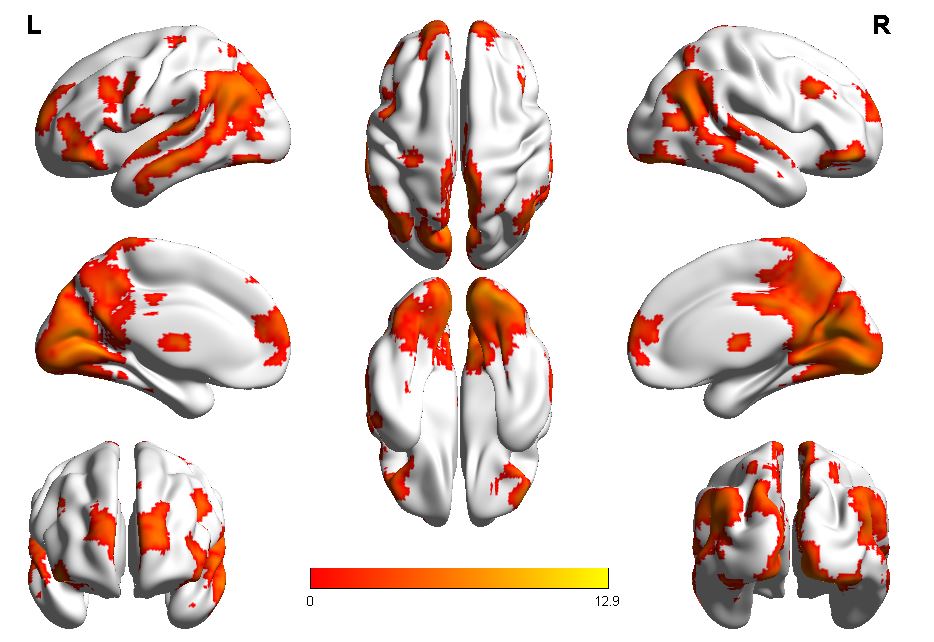


**Figure S3**. Functional nodes with large number of connections in vascular mild cognitive impairment group. The threshold was set at voxel level *p* < 0.05 corrected for multiple comparisons using the family-wise error rate. The color bar represents the t value of the within-group analysis. L, left; R, right.


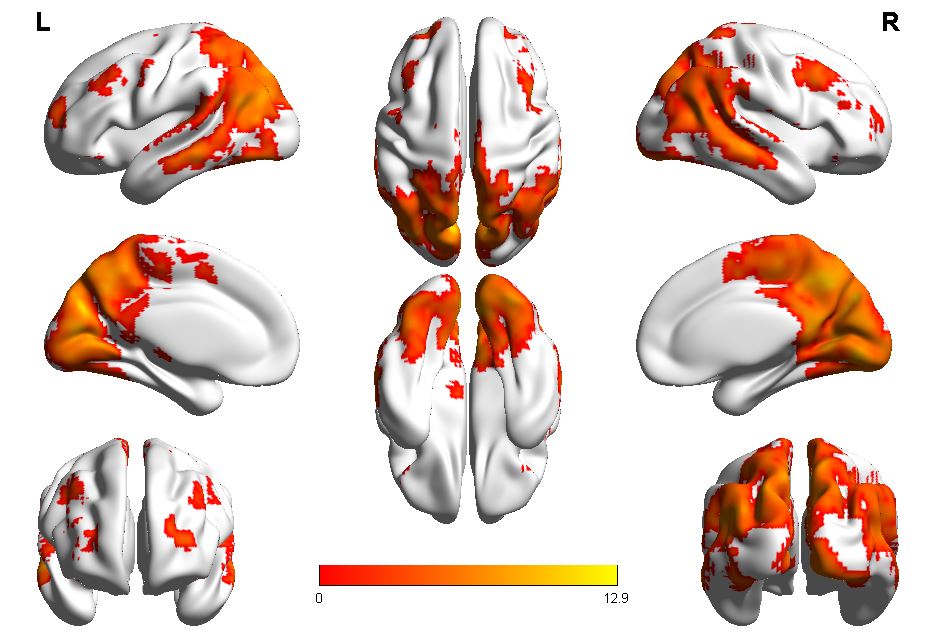


**Figure S4**. Whole brain functional connectivity of left middle temporal gyrus in health control group. The threshold was set at voxel level *p* < 0.05 corrected for multiple comparisons using the family-wise error rate. The color bar represents the t value of the within-group analysis. L, left; R, right.


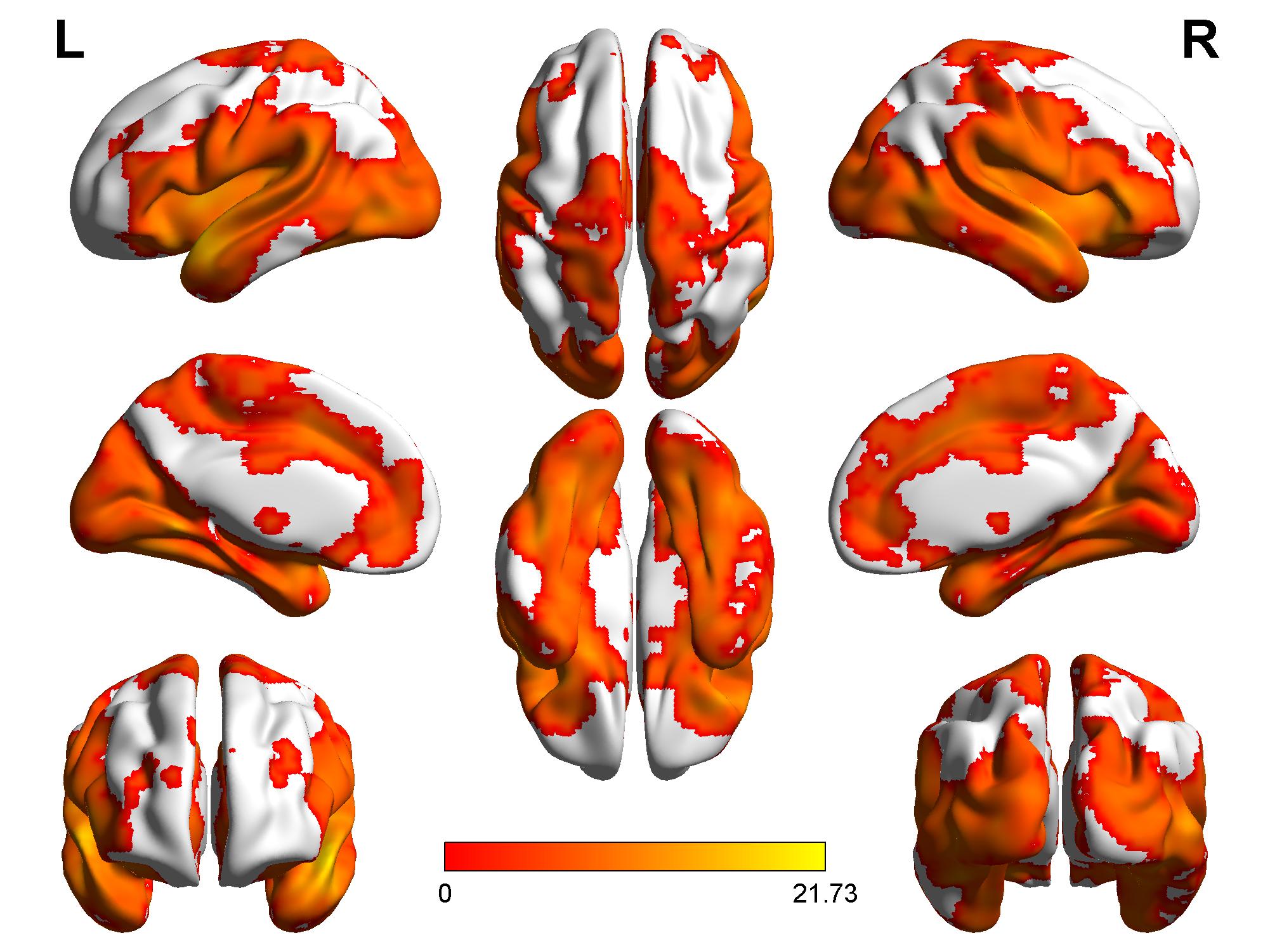


**Figure S5**. Whole brain functional connectivity of left middle temporal gyrus in amnestic mild cognitive impairment group. The threshold was set at voxel level *p* < 0.05 corrected for multiple comparisons using the family-wise error rate. The color bar represents the t value of the within-group analysis. L, left; R, right.


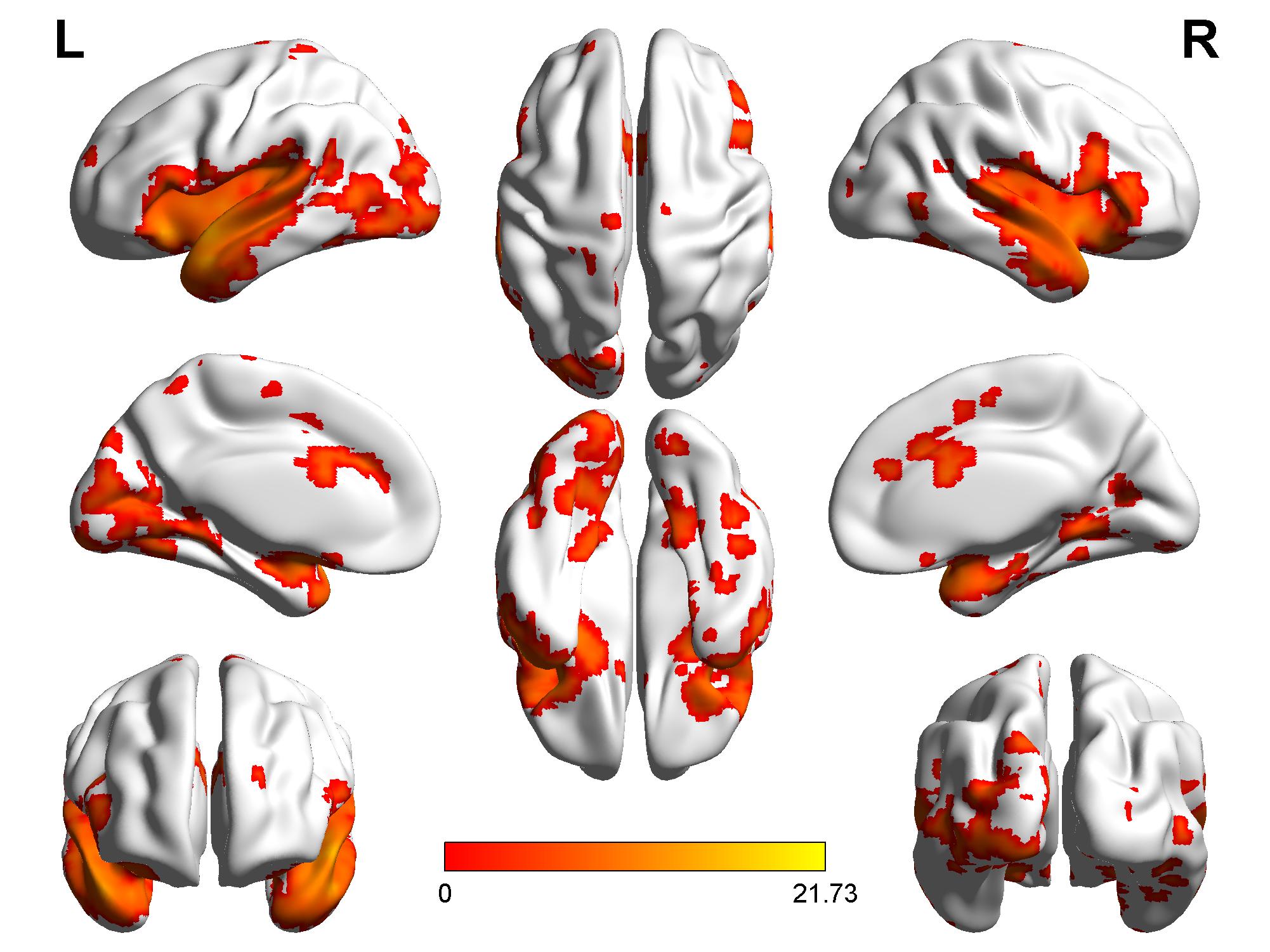


**Figure S6**. Whole brain functional connectivity of left middle temporal gyrus in vascular mild cognitive impairment group. The threshold was set at voxel level *p* < 0.05 corrected for multiple comparisons using the family-wise error rate. The color bar represents the t value of the within-group analysis. L, left; R, right.


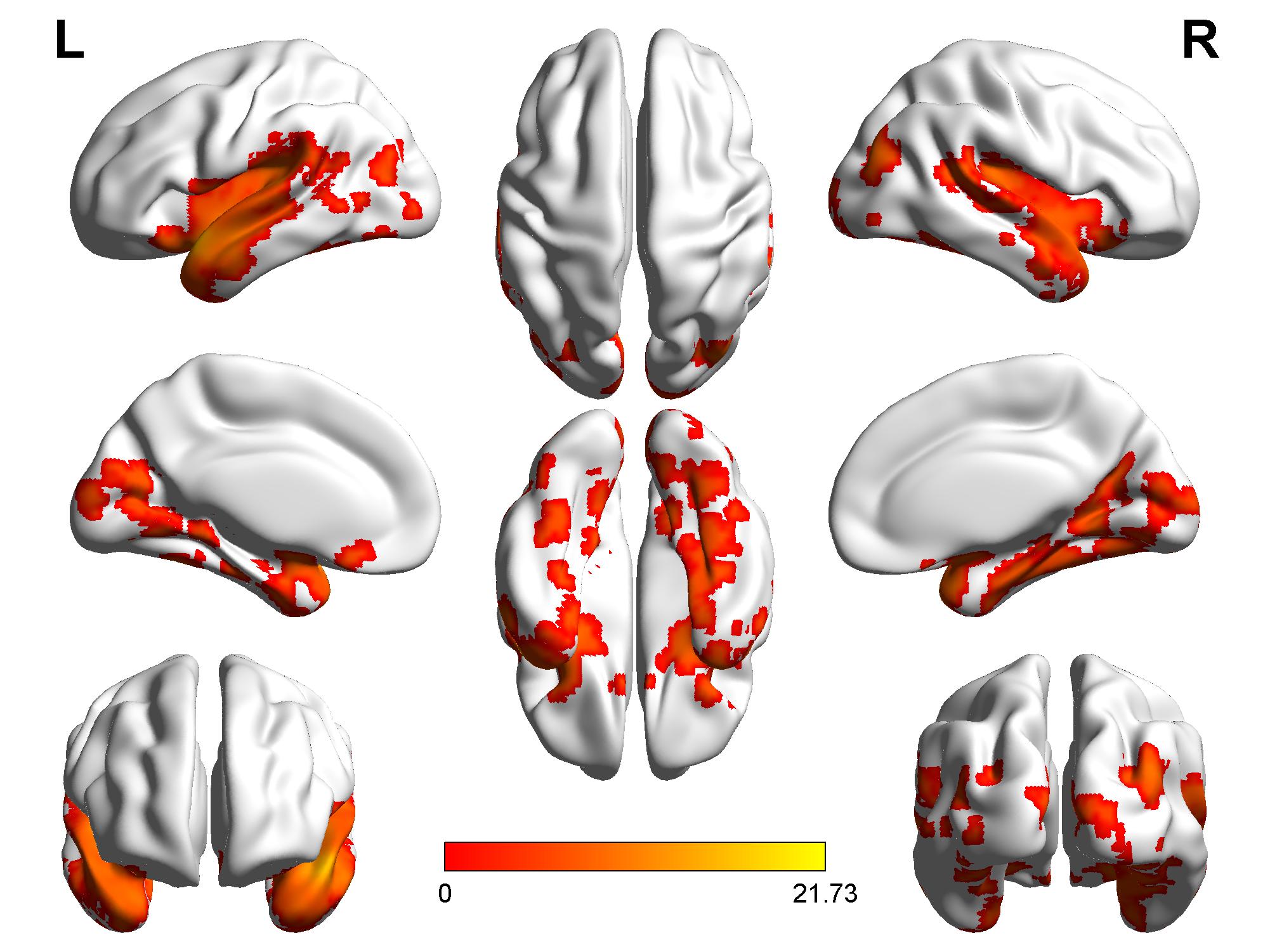

Supplement: Supplementary Materials — The attached file of supplementary materials presents the results derived from one-sample t-tests of within-group analysis for the three groups. Figures S1–S3 show the functional nodes with a large number of connections in HC, aMCI, and VaMCI, separately. Figures S4–S6 show the whole-brain FC of the left MTG in HC, aMCI, and VaMCI, separately. [file 8812490.f1.docx]
